# Development and Evaluation of Nanoformulations Containing Timur Oil and Rosemary Oil for Treatment of Topical Fungal Infections

**DOI:** 10.3390/gels9070516

**Published:** 2023-06-26

**Authors:** Afeefa Noor, Shahid Jamil, Tariq Waece Sadeq, Muath Sheet Mohammed Ameen, Kanchan Kohli

**Affiliations:** 1 Lloyd Institute of Management and Technology, Greater Noida 201306, India; 2 Department of Pharmaceutics, School of Pharmaceutical Education and Research (SPER), Jamia Hamdard University, New Delhi 110062, India; 3 Department of Pharmacy, College of Pharmacy, Knowledge University, Kirkuk Road, Erbil 44001, Iraq; 4 Pharmacy Department, Erbil Medical Technical Institute, Erbil Polytechnic University, Ebril 44001, Iraq

**Keywords:** Timur oil, rosemary oil, nanoemulgel, fungal infections

## Abstract

The pervasiveness of fungal infections is an issue for skin health globally, and there are a reported 40 million cases in developed and developing countries. Novel drug delivery systems provide better therapeutic efficacy over conventional drug therapy due to their lower side effects and toxicity. Furthermore, combinations of essential oils can represent alternative therapies for fungal infections that are resistant to synthetic drugs. This study is aimed at developing Timur oil into a nanoemulgel and evaluating its antifungal effects. The development of the formulation involved the preparation of a nanoemulsion by the titration method, followed by its evaluation for various physicochemical properties. The antifungal activity of the nanoemulgel against *Candida albicans* was evaluated. The zone of inhibition was determined using the disk diffusion method. The results show that the developed nanoemulgel has a particle size of 139 ± 6.11 nm, a PDI of 0.309, and a zeta potential of −19.12 ± 2.73 mV. An in vitro drug release study showed a sustained release of 70 ± 0.289% of the drug over a period of 24 h. The % drug permeation across the skin was found to be 79.11 ± 0.319% over 24 h. However, the amount of drug retained in the skin was 56.45 µg/g. The flux for the nanoemulgel was found to be 94.947 µg/cm^2^/h, indicating a better permeability profile. The nanoemulgel formulation showed a zone of inhibition of 15 ± 2.45 mm, whereas the 1% ketoconazole cream (marketed preparation) exhibited a zone of inhibition of 13 ± 2.13 mm. The results of this study suggest that developed nanoemulgel containing Timur oil and rosemary oil has the potential to be used for treating topical fungal infections caused by *Candida albicans*.

## 1. Introduction

Topical fungal infections greatly affect skin health in developing and developed countries. *Candida* species are the most common fungi for superficial skin infections. Fungal infections infect the skin superficially and then enter the deeper layers of the skin by desquamation; hence, agents that can permeate deep into the skin are needed to treat these infections [1]. Topical therapies are the preferred treatment for dermal fungal infections because of their targeted action and reduced side effects [1,2]. Various synthetic drugs such as itraconazole, ketoconazole, and clotrimazole are used as conventional topical drugs for fungal infections. Although they have advantages such as localized effects, increased bioavailability, and good patient compliance, conventional drugs also have disadvantages, such as the potential to trigger allergies and eczema [1]. The repeated use of synthetic drugs also increases toxicity and resistance [3].

The utilization of essential oils to combat microbial infections is gaining importance. Timur oil (*Zanthoxylum armatum*), of the family *Rutaceae*, is an aromatic, annual herb, mostly growing in the hot valleys of the subtropical Himalayas, from Indus areas to Bhutan, up to an altitude of 2400 m. It also grows in the Khasi Hills from 700 m to 1000 m. Essential oils and fatty acids are thought to be the main constituents of this medicinal plant. The essential oils obtained from fruits of *Zanthoxylum armatum* have antifungal, antibacterial, and anthelmintic properties. Numerous scientific investigations have shown the antifungal [4] and mosquito-repelling properties [5] of Timur oil. According to Tiwary et al. [6], the main ingredients of the essential oil of *Zanthoxylum armatum* are linalool (57%) and limonene (19.8%). The linalool content in essential oils has been shown to mainly account for their antifungal properties [7]. However, limonene has modest antibacterial activity while also exhibiting effective antimicrobial action [4]. Prakash et al. [8] reported that the essential oil of a different variety of Zanthoxylum possesses a wide range of antifungal activity against molds such as *Aspergillus flavus*, *niger*, *terreus*, *candidus*, *sydowi*, *fumigates*, *Alternaria alternate*, *Cladosporium cladosporioides*, *Curvular ialunata*, *Fusarium nivale*, *Penicillium italicum*, and *Trichoderma virdie*. Zanthoxylum alatum showed a zone of inhibition 18 mm in diameter against *Alternaria alternate* [9].

Rosemary is a member of the mint family [10], and it possesses various antioxidants such as carnosic acid, carnosol, and rosmarinic acid [11,12]. Additionally, it has significant concentrations of flavonoids, phenolic acid, diterpene, and triterpene. Furthermore, studies have revealed that rosemary oil exerts antifungal [10], antibacterial, anti-inflammatory [13] antimicrobial [14], and antileishmanial activities [15]. The protective/beneficial effects of rosemary essential oil on candidiasis have been explored in a few studies [16].

Recent findings have revealed that natural herbal products can be used to treat major health issues such as cancer, microbial infections, and cardiovascular disorders. Therefore, novel drug delivery systems are required to tackle the challenges associated with these natural compounds to increase their effectiveness [17]. Nanoemulsion is a novel approach to producing stable preparations with increased solubility. Producing nanoemulsions is a good technique for developing formulations that contain essential oils as active ingredients for antifungal activity [18]. The spontaneous emulsification method (titration method) for nanoemulsion is very simple. It includes gently swirling while combining oil, water, surfactant, and co-surfactant in an ideal ratio [19]. To determine this ideal ratio, a phase diagram can be constructed using the aqueous titration technique. This method produces particles that range in size from 20 to 200 nm [20,21]. The hydrophilic character of the formulation makes it simple to remove the NEG from the application site when the necessary efficacy has been achieved. The thixotropic NEG facilitates easy spreadability at the target site and prolongs retention at the application site due to its mucoadhesive property [22,23]. In order to increase the thickness, lower the interfacial tension, and improve the stability, a nanoemulsion is added to the hydrogel matrix, such as a carbomer (Carbopol), to create a nanoemulgel, which functions as a drug reservoir [24]. The nanoemulgel increases the perforation of the oil into the skin [25]. When oily particles exit the skin’s gel matrix intact and enter its layers, they reach their bodily targets [26].

The study aimed to use a rosemary-mediated nanoemulgel containing Timur oil as the main oil to effectively treat topical fungal infections. In this study, Timur oil is nanoemulsified in Smix and rosemary oil is used as carrier oil on the basis of its miscibility with Timur oil. Considering geographical factors as important parameters for herbal products, the extraction of two varieties of Timur seeds from different geographical sources (India and Nepal) was carried out to determine the percentages of linalool in both varieties by GC analysis. As this combination was new, there has not yet been research on combining these two essential oils for antifungal activity. In this study, Tim–Ros–NEG is the developed rosemary-mediated nanoemulgel containing Timur oil and its topical antifungal activity was evaluated against *C. albicans*, compared with that of a 1% ketoconazole cream (marketed preparation).

## 2. Results and Discussion

### 2.1. Extraction of Timur Oil and Characterization

**Figure 1:**
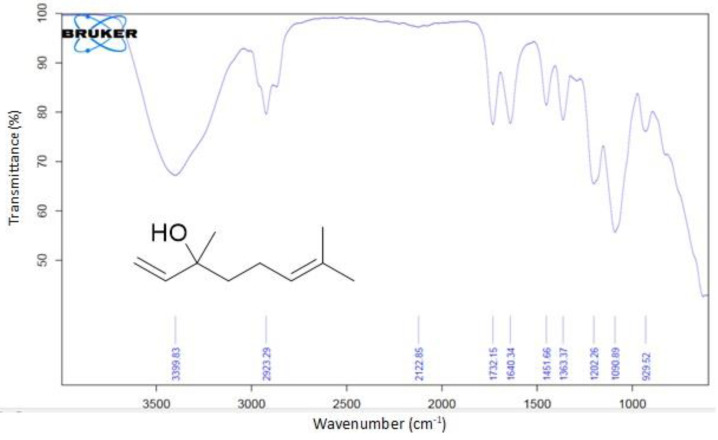
FT-IR Spectra of Timur oil and chemical structure of linalool.

**Figure 2:**
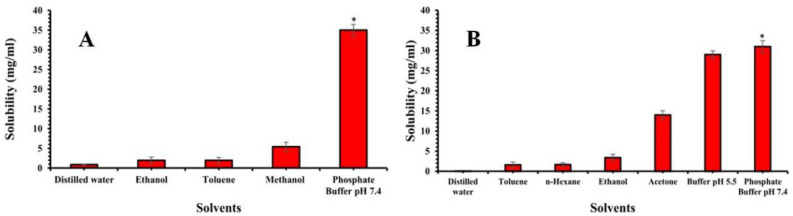
(**A**) Mean solubility (mg/mL) of rosemary oil at 25 °C. (**B**) Mean solubility (mg/mL) of Timur oil at 25 °C. An asterisk (*) indicates that the value is significantly different at *p* < 0.05 compared to the other group.

In order to develop a nanoformulation containing Timur oil and rosemary oil, Timur oil was extracted from the fresh seeds of *Zanthoxylum Armatum* (Timur) of Indian and Nepali origin, with a 56% percentage of active constituents (linalool) of Nepali origin; the yield was 1.5 percent/200 g. The Timur oil was characterized by FTIR, HPTLC, and GC. The FTIR functional group was identified, and the results are shown in Figure 1 and Table 1. The quantification of linalool was carried out by GC, and the area for linalool (Standard) and the area for linalool present in the Timur oil were found to be 272,960,345 and 51,550,363, respectively. The retention times for linalool (Standard) and linalool in the extracted Timur oil were observed to be 15.583 and 15.531 (Table 2), respectively. HPTLC analysis showed that the linalool content in the Nepali variety (56%) was higher than that in the Indian variety (36%). Therefore, the variety from Nepal was selected for further studies. In order to study the release of the developed formulation, solubility studies for Timur and rosemary oil were carried out in distilled water, methanol, ethanol, toluene, and phosphate buffer at pH values of 5.5 and 7.4. Figure 2 represents the maximum solubility of rosemary oil in buffer at pH 7.4, while Timur oil was soluble in acetone and buffer at pH 5.5 and 7.4. It was observed that the rosemary oil and Timur oil have significantly (*p* < 0.05) higher solubility in pH 7.4 phosphate buffer compared to the other solvents.

### 2.2. Construction of Pseudoternary Phase Diagram

**Figure 3:**
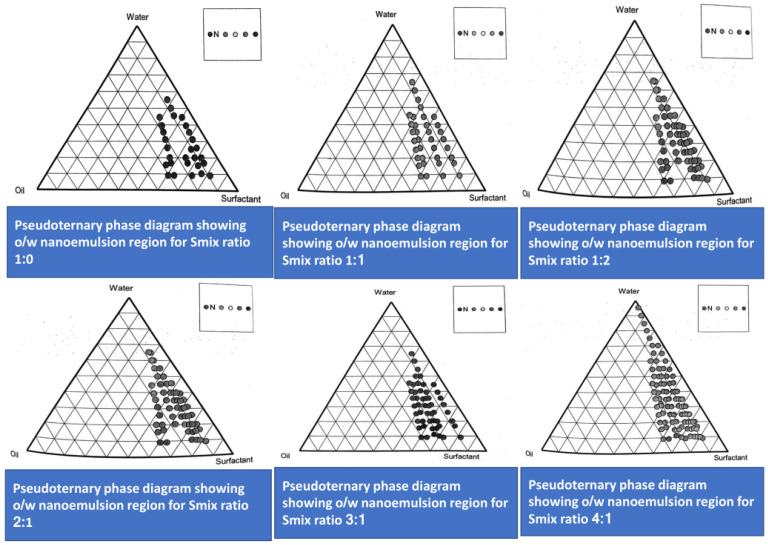
Pseudoternary phase diagram showing o/w nanoemulsion regions for the Smix ratio (1:0, 1:1, 1:2, 2:1, 3:1, and 4:1).

Timur oil, rosemary oil, Transcutol P (cosurfactant), and Tween 80 (surfactant) were combined at various concentrations to create ternary phase diagrams to identify the best formulation, which resulted in a nanoemulsion with a PDI of 0.3 and a droplet size of less than 200 nm (Figure 3). Pseudoternary phase diagrams were constructed by preparing various Smix ratios (1:0, 1:1, 1:2, 2:1, 3:1, and 4:1). First, placebo formulations were prepared using the selected oil/surfactant/co-surfactant in the above-mentioned Smix ratio. After the formation of the placebo, drug loading was performed for the placebo. Timur oil was used as the oil phase, Tween 80 as the surfactant, Transcutol P as the co-surfactant, and distilled water as the aqueous phase during phase diagram formation. The Smix ratio of 4:1 occupied the largest area out of all the ratios. Thus, it was determined that the 4:1 Smix ratio produced the largest possible nanoemulsion area.

An O/W nanoemulsion was prepared by using the titration method. It is well established that, the higher the quantity of the surfactant, the greater the potential for toxicity toward the skin and irritation. Therefore, an attempt was made to increase the oil concentration and decrease the concentration of the Smix. Therefore, from the titration chart, the suitable oil/Smix ratios were selected, which were 5:5, 6:4, and 7:3. Out of these, the 5:5 oil/Smix ratio was selected.

#### Physicochemical Characterization of Nanoemulsion

**Figure 4:**
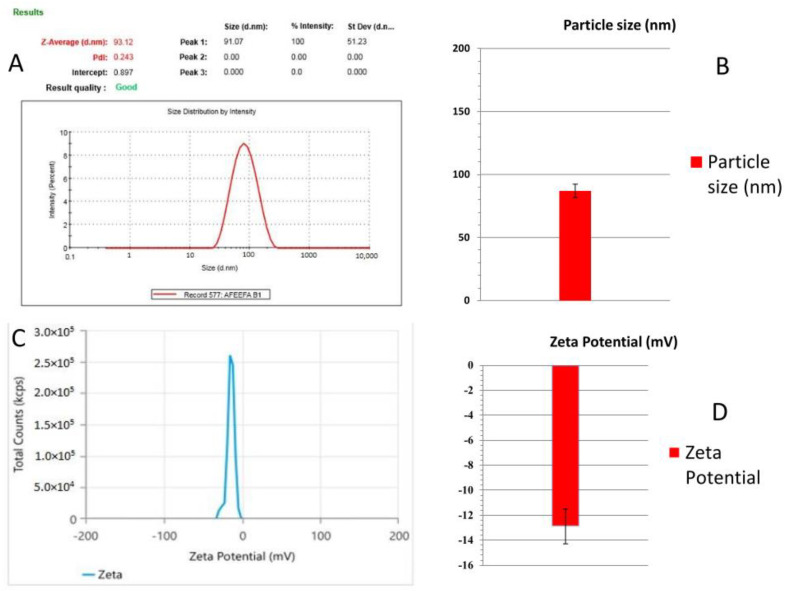
(**A**) Droplet size of developed nanoemulsion. (**B**) Bar diagram for droplet size of developed nanoemulsion. (**C**) Zeta potential of developed nanoemulsion. (**D**) Bar diagram for zeta potential of developed nanoemulsion.

The prepared nanoemulsion was examined for globule size using a Malvern Zetasizer [27]. The droplet size of the developed nanoemulsion (Smix ratio: 5:5) was 93.12 nm. In contrast, the polydispersity index of the same formulation was 0.243, which shows a better distribution of the globules of the dispersed phase into the dispersion system (Figure 4). The ideal range of the PDI lies between 0.1 and 0.5.

An appropriate zeta potential ensures that the nanoformulation system is intense and stable [27,28]. Nanoemulsions typically have a zeta potential value between +100 mV and −100 mV. Neutral nanoemulsions correspond to those with zeta potential values between −10 and +10 mV [29]. The developed nanoemulsion formulation showed a zeta potential value of −14.32 mv, indicating good stability (Figure 4).

### 2.3. Fabrication of Timur Oil Nanoemulgel Formulation

**Figure 5:**
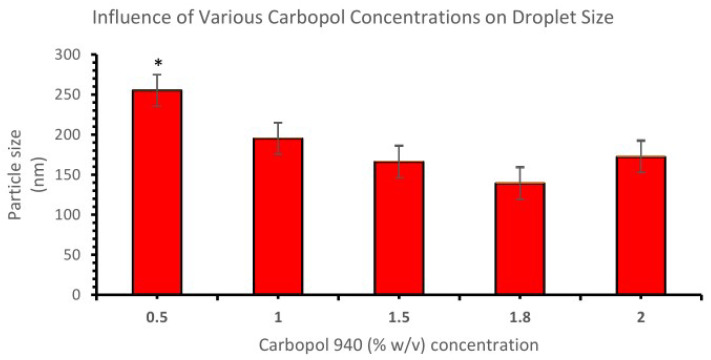
Influence of various Carbopol concentrations on droplet size. An asterisk (*) indicates that the value is significantly different at *p* < 0.05 compared to the other group.

Five nanoemulgel formulations were prepared using the same drug concentration but different concentrations of polymer (Carbopol-940), i.e., 0.5, 1.0, 1.5, 1.8, and 2.0% *w*/*v*, and the results were determined. It was found that, upon increasing the polymer concentration from 0.5% *w*/*v*, the particle size, as well as the polydispersity index of the respective formulations, decreased but only up to a polymer concentration of 1.8% *w*/*v*, and beyond this concentration, the particle size and PDI further increased (shown in Figure 5). Therefore, it was discovered that a Carbopol-940 concentration of 1.8% *w*/*v* was appropriate for creating an optimal nanoemulgel formulation. It was observed that the nanoemulgel containing 0.5% *w*/*v* polymer concentration significantly (*p* < 0.05) reduced the droplet size compared to the nanoemulgel containing 1%, 1.5%, 1.8% & 2% *w*/*v* polymer concentrations.

#### 2.3.1. Physicochemical characterization of Nanoemulgel

**Figure 6:**
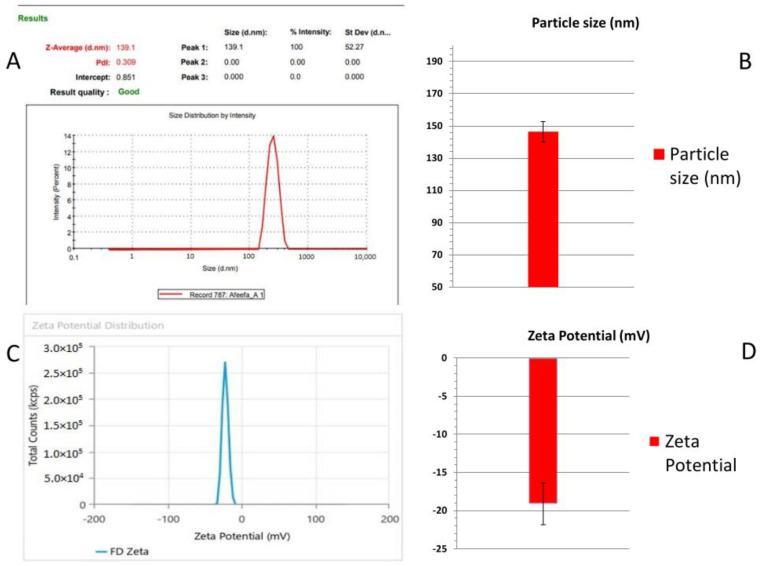
(**A**) Particle size of developed nanoemulgel. (**B**) Bar diagram for particle size of developed nanoemulgel. (**C**) Zeta potential for developed nanoemulgel. (**D**) Bar diagram for zeta potential of developed nanoemulgel.

The various nanoemulgel formulations were prepared, and their mean particle sizes were determined (Table 3 and Figure 6) [17]. The results revealed that the developed formulation (i.e., FD) showed a mean droplet size of 139 nm and a lower PDI. The value of the PDI should lie between 0.1 and 0.5. The developed formulation showed a PDI of 0.309, which shows a good size distribution for the droplets. 

This technique determines the charge on the globule surface of the dispersed phase. The zeta potential value of a nanoemulgel typically ranges from +100 mV to −100 mV [27,28,30,31]. The zeta potential of the developed nanoemulgel formulation was found to be −21.99 mV, −16.54 mV, and −18.85 mV, respectively. The mean zeta potential of the developed nanoemulgel formulation was observed as −19.12 ± 2.73 mV and we have shown these results in the bar chart (Figure 6D). We have considered a zeta potential value of −21.99 mV of the developed nanoemugel formulation (FD), indicating good formulation stability, as shown in Figure 6C.

#### 2.3.2. Mechanical Strength of NEG

**Figure 7:**
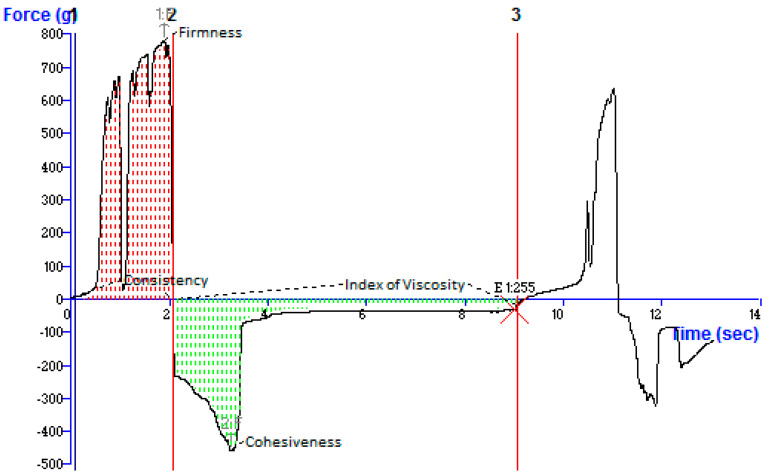
Texture Analysis of developed nanoemulgel showing the resultant force versus time.

Patient acceptability depends on creating a gel with the necessary physical characteristics, such as excellent pourability, spreadability, and acceptable hardness and viscosity. A texture analyzer was used to investigate the mechanical characteristics of Tim–Ros–NEG. The findings showed that the characteristics varied with the concentration of Carbopol 940 (0.5%, 1%, 1.5%, and 1.8%). With an increase in hydroxypropyl methylcellulose content, gel cohesiveness was observed to rise by Karavana et al. [32] and the same observation was recorded by Cevher et al. [33] for Carbopol gels. The developed nanoemulgel’s hardness was found to be 783.01 g force. Firmness or hardness refers to the formulation’s deformational flexibility under stress, while cohesiveness refers to the number of cross-links between gel molecules and their ability to maintain shape. The higher the value, the better the gel strength [23]. The findings demonstrated Tim–Ros–NEG’s resistance to deformation. However, our spreadability results demonstrated that Tim–Ros–NEG is spreadable even though it is more resistant to deformation. Typically, increased hardness results in less spreading. The developed Tim–Ros–NEG was determined to have a consistency of 898.34 g.s. The term “consistency” refers to a product’s viscosity and characterizes its texture and hardness. A high consistency score suggests that Tim–Ros–NEG was consistent, according to the investigation. Cohesiveness is just a measure of how effectively a product resists a second deformation in comparison to how well it resists a first deformation. The greatest force represents a sample’s stickiness or adhesiveness. According to the findings of our research, Tim–Ros–NEG is more cohesive or sticky than other materials since it demonstrated high cohesiveness (−459.81 g force) and an index of viscosity of 688.84 g/s. Higher Tim–Ros–NEG adhesion translates into a longer skin contact duration, which might result in less frequent nanoemulgel application. Osmałek et al. reported that Opokan^®^, a commercial product, had the lowest textural characteristics with a hardness of more than twice that of other gels [34]. Mohapatra et al. reported that Black Cohosh loaded ethosomal gel for the treatment of menopause. They have used Carbopol^®^ 971P NF as a gelling agent. They have considered firmness, consistency, cohesiveness, and work of cohesion as evaluation parameters [35]. Hasan et al. developed combined therapy of 5-fluorouracil (5-FU) and cannabidiol (CBD)-loaded nanostructured lipid carrier gel (FU-CBD-NLCs gel) for the effective treatment of non-melanoma skin cancer. In this study, they have reported textural properties of 5-fluorouracil (5-FU) and cannabidiol (CBD)-loaded nanostructured lipid carrier gel (FU-CBD-NLCs gel). They have used Carbopol 934 as a gelling agent. The results of our investigation are in accordance with the published research article [36]. The findings of FD’s texture analysis (Table 4 and Figure 7) indicated that the formulation was sufficiently firm and cohesive, showing that it would be user-friendly during application. Both formulations showed thermosensitivity, as indicated by a negative index of viscosity values.

#### 2.3.3. In Vitro Release Study

**Figure 8:**
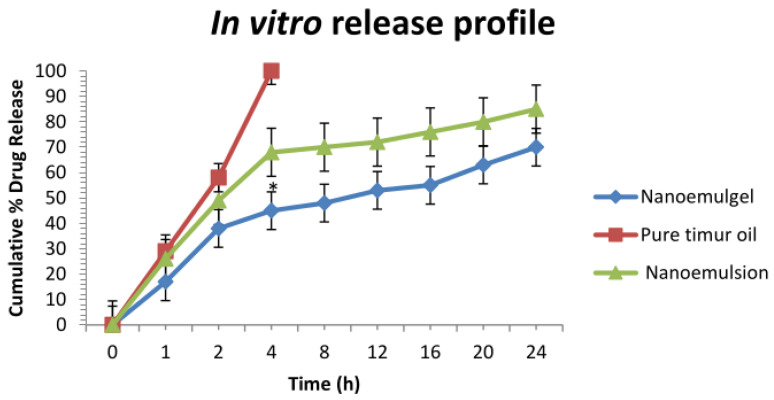
Comparison of in vitro release profile between pure Timur oil, nanoemulsion, and nanoemulgel. An asterisk (*) indicates that the value is significantly different at *p* < 0.05 compared to the other group.

The cumulative % release experiments of the Timur oil from developed nanoemulgel formulation were determined using phosphate buffer (with pH 5.5) and compared with that of the prepared nanoemulsion and pure Timur oil. Due to the essential oil’s encapsulation in the lipid portion of the nanoemulgel, the essential oil from the developed nanoemulgel was released slowly over time. Within 24 h, the developed nanoemulgel formulation showed a considerable release (70%) while the developed nanoemulsion formulation exhibited an 85% release (Figure 8). A 100% release was seen in the case of pure Timur oil within 4 h [3].

Any drug’s therapeutic effectiveness depends on how readily it is released from its pharmaceutical formulation [37]. The viscosity, surfactant content, polymer, and drug concentration are only a few of the variables that affect how quickly a topical pharmaceutical formulation releases a drug [38]. The development of a viscous formulation due to the synthetic polymer Carbopol 940′s large molecular weight delays the release profile of pharmaceuticals from topical preparations like nanoemulgel [39]. Additionally, it has been stated previously that emulgel serves as a reservoir for the drug, releasing it first from the internal phase to the exterior phase before entering the skin [40]. In contrast to nanoemulsion, which requires the oil droplets to pass through a hydrophilic phase transition, nanoemulgel allows the oil droplets to first be released into the gel matrix and then they can pass directly into the skin’s subcutaneous layer [41]. Additionally, among all the formulations, the nanoemulgel showed a superior sustained release effect, as shown in Figure 8 [42]. Therefore, based on this study, it was concluded that the Timur oil release with the developed formulation having a polymer concentration of 1.8% *w*/*v* was considerably higher, with a sustained release, compared to the pure Timur oil. Therefore, based on this study, it was concluded that among all the prepared nanoemulgel formulations, (FD) was the optimal formulation and can be considered for further in vivo studies. The antimicrobial action is due to the oil components and the drug delivery system, we used. The developed nanoemulgel formulation showed a significantly (*p* < 0.05) higher release of Timur oil compared to the in vitro release of pure Timur oil.

#### 2.3.4. Release Kinetics

**Figure 9:**
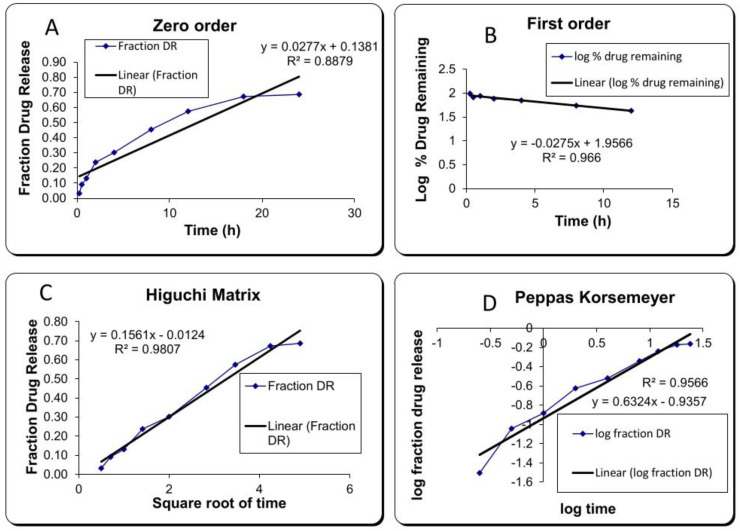
Various release kinetic models of the nanoemulgel: (**A**) zero-order kinetics; (**B**) first-order kinetics; (**C**) Higuchi matrix; (**D**) Korsmeyer–Peppas.

The release data were examined to determine the goodness of fit in various kinetic models in order to establish a suitable Tim–Ros–NEG release pattern. The release data were run through various release kinetic models, including zero-order, first-order, Higuchi matrix, and Korsmeyer–Peppas models, and the best-fitting model was chosen based on the regression coefficient (R^2^) value. The highest R^2^ value was 0.9807, while the lowest R^2^ value was 0.8879. The Higuchi matrix model was the best-fitting model for the nanoemulgel, having an R^2^ value of 0.9807 (Figure 9) [43]. The value of *n* was found below 0.5 (i.e., 0.1561), showing that Timur oil release from the developed nanoemulgel formulation follows Fickian diffusion [44].

#### 2.3.5. Drug Deposition Study

The skin retention properties of the nanoemulgel were determined using a drug deposition study. The skin retention of the Timur oil from the fourth nanoemulgel formulation (with the formulation code FD) was found to be 56.45 µg of the dose applied, whereas pure Timur oil was found to be 34.56 µg of the dose applied on the skin. The gel used for topical application has to have the right formulation properties to make it easy to apply and remain in contact with the skin for a long time. Tim–Ros–NEG at 1.8% had a greater viscosity and remained stuck to the skin for longer compared to Tim–Ros–NEG at 0.5%, 1.0%, and 1.5%. This nanoemulgel showed greater drug localization in the skin. Therefore, the nanoemulgel (FD) formulation resulted in more medication being retained in the skin, as demonstrated in Table 5.

#### 2.3.6. Morphological Studies/Internal Composition Study

**Figure 10:**
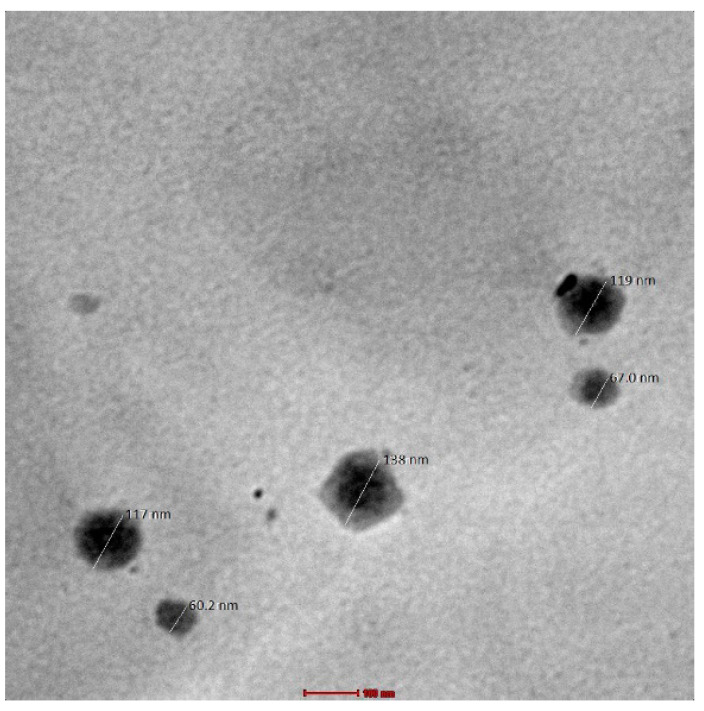
TEM of developed nanoemulgel (scale bar = 100 nm; 92,000× frame).

TEM is the most effective characterization technique that uses high magnification to anticipate morphological characteristics and examine samples’ surfaces [31,45,46]. TEM was used to study the morphology of the globules of the developed nanoemulgel. The globule size was in accordance with the results obtained using the Zetasizer. TEM images of the developed formulation (having the formulation code FD) indicated spherical globes with slight roughness, and no aggregation was observed among the globules (Figure 10).

#### 2.3.7. Confocal Laser Scanning Microscopy

**Figure 11:**
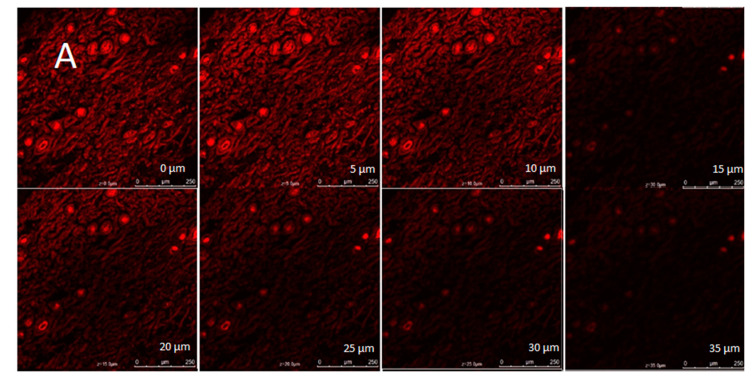

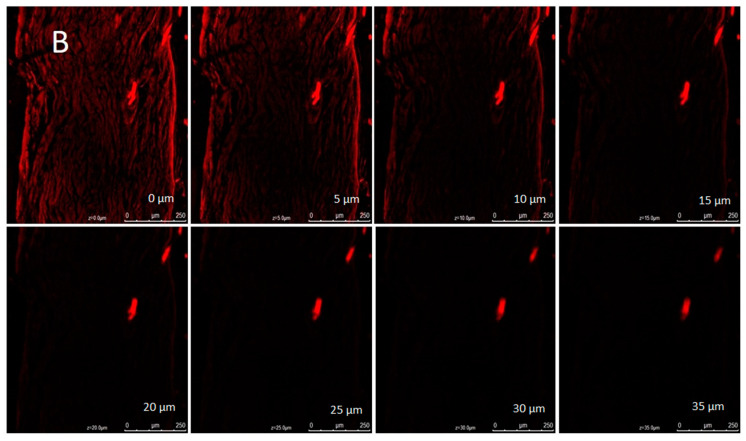

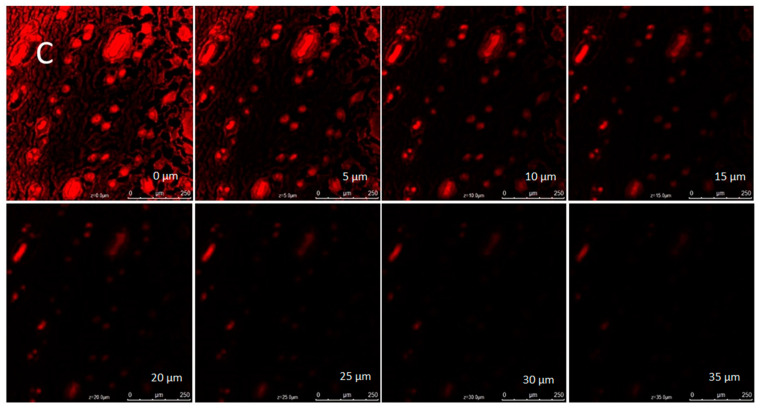

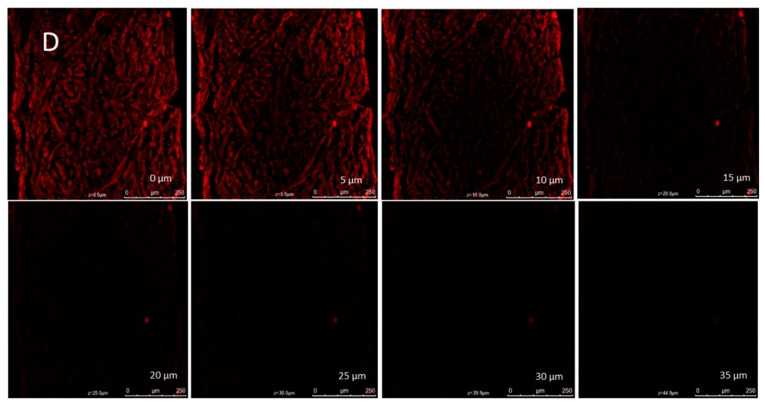
Confocal laser scanning microscopy images of excised rat skin treated with (**A**) plain dye solution, (**B**) dye-loaded marketed gel, (**C**) dye-loaded developed nanoemulsion, and (**D**) dye-loaded developed nanoemulgel formulation.

The CLSM investigation evaluated the depth and intensity of permeation through the skin of the rats treated with a marker-loaded commercial gel, the developed nanoemulsion, and the developed nanoemulgel. The outcomes were contrasted with those for the skin from the group that received the control treatment. The breadth and depth of the carrier system’s penetration into the skin were similar to the intensity and depth of the marker’s penetration. Figure 11A–D display the photomicrographs of rat skin treated with the control, commercial gel, developed nanoemulsion, and optimal nanoemulgel formulations. Compared to the control group, the treatment group treated with nanoemulgel-based formulations showed dramatically enhanced depth and intensity of penetration into the skin. This showed that formulations based on developed carriers have more potential for skin penetration.

#### 2.3.8. Ex Vivo Skin Permeation Study

**Figure 12:**
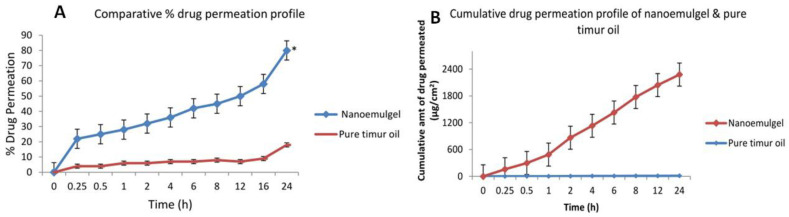
(**A**) Comparative % drug permeation profiles of the drug nanoemulgel and pure Timur oil. (**B**) Cumulative drug permeation profile of the nanoemulgel and pure Timur oil. An asterisk (*) indicates that the value is significantly different at *p* < 0.05 compared to the other group.

In order to compare the permeability of the nanoemulgel with that of a pure Timur oil, ex vivo permeation tests were carried out. Franz diffusion cells and rat skin were used in the study. Figure 12 shows the outcomes for the ex vivo permeation of Timur oil from the Tim–Ros–NEG and pure Timur oil. The Tim–Ros permeation from the two formulations is compared, and it is shown that the permeation of Timur oil from the created NEG formulation (2265.01 ± 0.01 µg/cm^2^) was considerably greater (*p* < 0.05) than that from the pure Timur oil (12.96 ± 0.33 µg/cm^2^) after 24 h (Figure 12). For the Tim–Ros–NEG, the % drug permeation for the nanoemulgel was found to be 21.7% at 0.5 h, 26.42% at 2 h, 44.73% at 6 h, 57.89% at 12 h, and 79.11% at 24 h, whereas the % DP of the pure Timur oil was 4.08% at 0.5 h, 5.71% at 2 h, 5.89% at 6 h, 12% at 12 h, and 15.03% at 24 h (Figure 12). The nanosized oil globules possessing Timur oil enhance the penetration, and may accelerate drug permeation through the skin’s lipophilic layers [47,48,49]. As the permeation parameters were examined simultaneously, it was discovered that the permeation flux was considerably increased (*p* < 0.05) with Tim–Ros–NEG (484.12 ± 0.065 µg cm^−2^ h^−1^) compared to the pure Timur oil (3.21 ± 0.074 µg cm^−2^ h^−1^). The nanoemulgel showed a better permeability profile, as the flux was 94.947 µg/cm^2^/h, whereas the flux of the pure Timur oil was 24.504 µg/cm^2^/h, much lower than that of the nanoemulgel. The nanoemulgel permeability was four times better than that of the pure Timur oil. When comparing the apparent penetration and flux of the two formulations, it was discovered that there was a four-fold augmentation ratio between the Tim–Ros–NEG and the pure Timur oil. This could be because the skin permeability increased due to Carbopol-940 [50], and the nanometric lipophilic globules also made this possible.

#### 2.3.9. Skin Irritation Study

**Figure 13:**
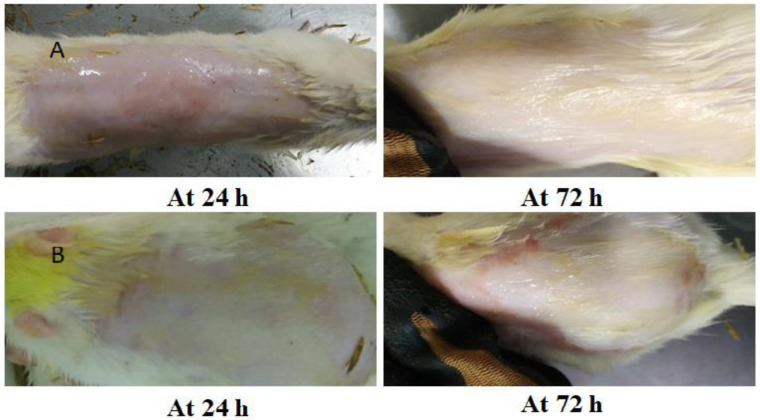
(**A**) Irritation study on nude rat skin conducted by applying the developed nanoemulsion. (**B**) Irritation study on nude rat skin conducted by applying the developed nanoemulgel.

A skin irritancy test was performed to verify the safety of the gel formulation. The formulation exhibited no erythemal or edematous scores even after 72 h. The average skin irritation score was 0 (no erythema in a rat), which is less than 5, demonstrating that the gel does not irritate Wistar rat skin when applied to it (Figure 13) [20]. Table 6 presents the results of this study. According to Draize et al. (33), a primary irritancy index (PII) value of less than two (2) denotes that the applied formulation is not irritating to human skin. As a result, because the PII for optimised nanoemulgel and optimised nanoemulsion was less than 2, they were considered to be non-irritants. Developing an appropriate nanoemulgel formulation depends heavily on the choice of surfactant. To compare ionic and nonionic surfactants, Tween 80, Tween 20, Span 80, polyethylene glycol 400, and PEG 200 (a nonionic surfactant) were chosen to develop the Tim–Ros–NEG. These nonionic surfactants generate homogeneous, superior droplets with a low critical micelle concentration, which aids in the quick absorption and release of the nanoemulgel. They also have a minimal risk of causing irritation and show low toxicity compared to other materials [19]. Therefore, it was determined that all the excipients included in the formulation were non-irritating and safe for topical usage.

#### 2.3.10. Antifungal Activity

**Figure 14:**
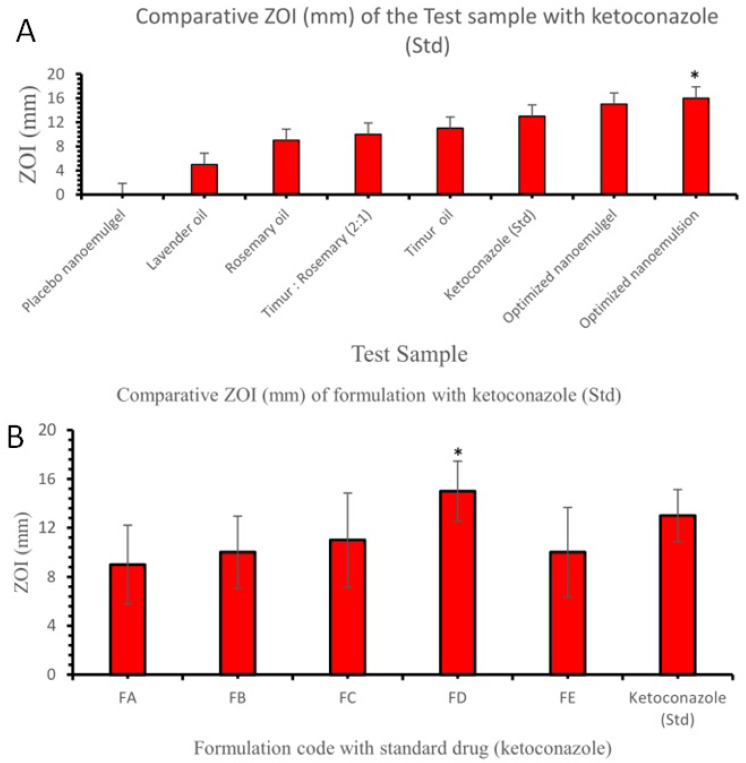
(**A**) Comparison of the ZOIs (mm) of the test samples with the ZOI of ketoconazole (Standard drug). (**B**) Comparison of the ZOI (mm) of the nanoemulgel formulation with that of ketoconazole (Standard drug). An asterisk (*) indicates that the value is significantly different at *p* < 0.05 compared to the other group.

**Figure 15:**
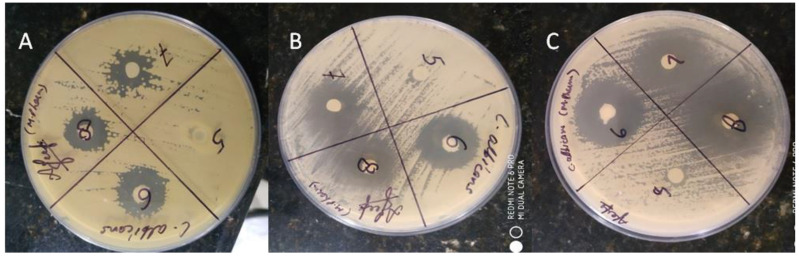
(**A**) ZOIs (mm) of the developed Smix (5), Timur oil (6), lavender oil (7), and rosemary oil (**8**) against *C*. *albicans.* (**B**) ZOIs (mm) of the placebo nanoemulgel (5), Timur (2): rosemary (1) (6), Timur oil (7), and developed nanoemulsion (8). (**C**) ZOIs (mm) of the placebo nanoemulsion (5), ketoconole standard (6), Timur (2): rosemary (1) (7), and 1.8% nanoemulgel (8) against *C. albicans*.

The results for the in vitro antifungal efficacy of Smix, Timur oil, lavender oil, rosemary oil, ketoconazole (standard), Timur:rosemary (2:1), the developed nanoemulsion, and a 1.8% nanoemulgel obtained using the cylinder plate method are presented in Figure 14 and Figure 15, and in Table 7. The antifungal activity of each sample or formulation was assessed at a concentration of 10 µg/mL (Table 7). It was found that the developed (Tim–Ros–NEG) formulations exhibited promising antifungal activity against *C. albicans*. It was revealed that the Tim–Ros–NEG significantly suppressed the growth of *C. albicans* compared to the other test preparations.

The developed nanoemulsion formulation was selected for the fabrication of the nanoemulgel with different Carbopol Ultrez 21 and Carbopol 940 concentrations (viz., 0.5%, 1%, 1.5%, 1.8%, and 2% gels). The developed Tim–Ros–NEG formulation was notably effective as an antifungal agent.

Timur oil exhibited an inherent antifungal effect reflected by the zone of inhibition for the developed nanoemulsion. Due to the synergistic effects of Timur oil and rosemary oil when delivered together using nanoemulsion technology, which permitted the intensive diffusion of the drug-containing oil globules, Tim–Ros–NEG’s action was enhanced. Timur oil, lavender oil, Timur:rosemary (2:1), and the developed nanoemulsion produced results comparable to those for the known antifungal essential oil and the reference drug (ketoconazole). The Tim–Ros nanoemulsion showed statistically significant growth suppression [51]. As a result, it was discovered that the nanoemulsion platform using Timur oil as the oil core had superior efficacy against *C. albicans* species. When both Timur oil and rosemary oil were used in combination, better antifungal activity than ketoconazole against *C. albicans* was observed. Timur oil and rosemary oil were combined in different ratios, and they gave comparable results when compared to the reference drug (ketoconazole). Both Timur oil and rosemary oil have antifungal activity themselves. When we used a 2:1 ratio of Timur:rosemary oil, the oils showed more synergistic activity compared with the other ratios of Timur oil and rosemary oil, as described in Table 7 and Figure 14. It was apparent that Tim–Ros nanoemulsion and Tim–Ros–NEG were active against *C. albicans*, and showed a significantly greater zone of inhibition compared to that resulting from placebo NEG and the marketed formulation (*p* < 0.05). 

#### 2.3.11. Histopathological Study

**Figure 16:**
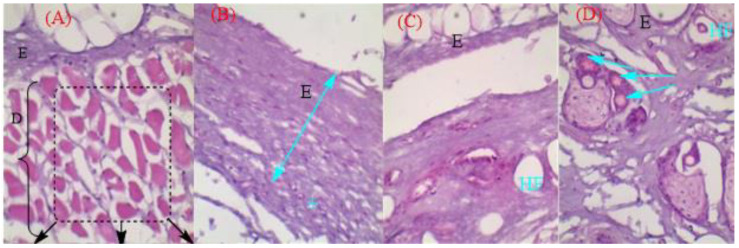
Histological micrographs of hematoxylin-eosin-stained skin section showing the normal epidermis, dermis tissues of (**A**) untreated rat skin, (**B**) formalin-exposed rat skin showed thickened degenerated epidermis E and inflammatory cells infiltrate(+). (**C**) Rat skin treated with conventional oil (Rosemary oil). (**D**) Rat skin treated with nanoemulgel enriched with Timur oil and Rosemary oil. The florescent blue arrow indicates blood vessel. E: Epidermis; D: Dermis; HF: Hair follicle.

Histopathological analysis was carried out to evaluate the toxicity profile of the developed formulation (Tim–Ros–NEG). The animal groups were divided into four groups: normal control, formalin solution treatment (positive control), blank NEG, and Tim–Ros–NEG. The rat skin was observed in positive control, blank NEG, and Tim–Ros–NEG groups and was compared with the control group. A histopathological examination of skin from the rat’s dorsal skin was performed to look for any indications of inflammatory reactions. Pathological alterations, including a thicker, deteriorated epidermis (E), intercellular edema, and inflammatory cell infiltrate were visible in the formalin-treated group (Figure 16). The Tim–Ros–NEG-treated groups did not exhibit aberrant alterations in the treated rat skin tissue compared to controls, other than a slightly thicker epidermis. Overall, the findings showed that the Tim–Ros–NEG was safe for topical administration and within the skin’s tolerance limit. No macrophages or lymphocytes were observed on the rat’s dorsal skin, indicating that there was no major inflammation in the surrounding tissues (Figure 16). This suggests that Tim–Ros–NEG might be safely administered topically using the formulated nanoemulgel.

## 3. Conclusions

In the present study, the topical application of a Timur–rosemary oil nanoemulgel (Tim–Ros–NEG) showed antifungal activity against *C. Albicans* count, improving its effect compared to the action of pure Timur oil. Moreover, we observed a 15 ± 2.9 mm ZOI from the Timur oil:rosemary oil (2:1) nanoemulgel in comparison to the pure Timur oil (11 ± 0.7 mm) and standard drug ketoconazole (13 ± 0.8 mm). The Timur oil:rosemary oil (2:1) nanoemulgel exhibited a synergistic effect. We believe that, if we used it in combination with ketoconazole, it would also have a synergistic effect. Therefore, we could reduce the therapeutic dose of ketoconazole, thereby reducing the toxicity caused by ketoconazole.

Natural substances such as Timur oil and rosemary oil have been considered to be suitable options for topical and even oral treatments because synthetic drugs can have undesirable effects and some infections caused by *Candida* are resistant to them. It was possible to create a nanoemulgel by adding Carpobol 940, a hydrogel material, at a concentration of 1.8% to a nanoemulsion containing Timur oil, rosemary oil, Tween 80, and Transcutol P. This method allows for greater penetration through the skin, improving the topical bioavailability and increasing the retention time.

When Timur oil and rosemary oil were added to the mixture, the tested microbial strains were suppressed more effectively due to better penetration of the oil globules from the NEG. The developed formulation was confirmed to be safe when tested on the skin of Wistar rats. The animals treated with Tim–Ros–NEG showed no indications of erythema. Additionally, the histopathological investigation showed no toxicity on the skin of the Wistar rats, indicating that the formulation is safe and effective for topical use. Consequently, the data suggest that the use of Tim–Ros–NEG might be a promising approach for the safe, highly effective, targeted delivery of Timur oil.

## 4. Materials and Methods

### 4.1. Materials

Tween 80, Carbopol 940, Carbopol Ultrez 21, acetone, chloroform, propylene glycol, disodium hydrogen phosphate, and Span 80 were obtained from S D Fine Chemicals Ltd., Mumbai, India. Methanol was purchased from Merck, Mumbai, India. Mueller–Hinton agar (which is produced by Becton, Dickinson, and company, Franklin lakes, NJ, United States) was used in the culture media. Marketed Timur oil was purchased from Moksha lifestyle, New Delhi (India). The rosemary oil and lavender oil were obtained from Pharmacos, Faridabad, Haryana, India. The Indian variety of Timur seeds and Nepali variety of Timur seeds were obtained from Hari Gokul, Khari Baoli, Old Delhi, India, and Khopra, Nepal, respectively. Both Indian and Nepali varieties of Timur seeds, authenticated by Prof. Javed Ahmad, Ex-head Department of Botany, School of Chemical & Life Sciences, Jamia Hamdard, New Delhi, has been deposited in the Herbarium of the herbal garden, Jamia Hamdard, New Delhi.

### 4.2. Extraction of Timur Oil from Timur Seeds

A total of 200 g of Timur seeds were weighed and crushed using a mortar and pestle. Then, the crushed Timur seeds were transferred to the clean round-bottomed flask of the Clevenger apparatus setup. A hydro-distillation process lasting 6–7 h was conducted. After completion of the distillation process, the Timur oil was collected. The collected Timur oil was dried using sodium sulfate to absorb the excess water. Then, it was filtered and the obtained oil was stored at 2 °C to 7 °C [52].

#### 4.2.1. Identification of Timur Oil 

##### Fourier Transform Infrared (FTIR) Spectroscopy

A Perkin–Elmer 591B spectrophotometer was used to determine the FTIR spectra of the Timur oil and Rosemary oil with KBr pellets or Nujol films, and scanning was performed at wavelengths of 4000 to 400 (cm^−1^) to obtain the characteristic spectra.

##### HPTLC Analysis for Qualitative Estimation of linalool in Timur Oil (Indian Variety and Nepali Variety)

The separate applications of each oil were made on 5 cm × 10 cm chromatographic precoated silica gel plates (Merck, TLC grade) that served as the stationary phase. Toluene and ethyl acetate (95:5 *v*/*v*) were used as the mobile phase in a twin-trough glass chamber where the TLC plates were prepared. The plates were taken off after the solvent front had moved 15 cm away from the initial extract site, and they were then left to dry. When the spots on the produced plates had dried, they were examined under visible (white), short UV (254 nm), and long UV (366 nm) light.

##### GC Analysis of Timur Oil, Marketed Timur Oil, and Nepali Timur Oil for Quantification of Linalool Content

The oils were subjected to GC analysis on a Shimadzu 15A gas chromatograph with a split/splitless injector (250 °C). The DB-5 capillary column (30 m 0.25 mm, film thickness 0.32 μm) was used, and nitrogen was utilized as the carrier gas (1 mL min^−1^). After maintaining a temperature of 60 °C for three minutes, the column was heated to 220 °C at a rate of 5 °C min^−1^ and maintained at this temperature for five minutes. The relative percentage quantity was estimated using a Shimadzu C-R4A Chromatopac from the peak region [53].

### 4.3. Screening of Components for Preparation of Nanoemulsion

The miscibility of the chosen oil served as the criterion for choosing the surfactant and co-surfactant. To make the stable nanoemulsion, the combination of a high HLB value with a low HLB value at the optimum temperature was used. Miscibility studies with oil were carried out by mixing Smix in a 5:5 ratio and vortexing for 5 min. Then, the mixtures were kept at room temperature for 24 h. After 24 h, an evaluation was performed based on the color change and phase separation [54].

### 4.4. Construction of Pseudoternary Phase Diagram

After the selection of the surfactant and co-surfactant (Smix) based on the miscibility with the oil, Smix was mixed in different volume ratios (1:0, 1:1, 1:2, 1:3, 2:1, 3:1, and 4:1). Based on the titration chart, different ratios of Smix were developed. Based on the points obtained from the titration chart, the phase diagram was made. The phase diagram was studied in detail for the formulation of the nanoemulsion [54].

### 4.5. Method for Preparation of the Phase Diagram (Aqueous Titration Method)

For the preparation of the phase diagram, the oil and different volume ratios of Smix were mixed. The ratios ranged from 1:9 to 9:1, and these were mixed to make 16 different combinations, which were 1:9, 1:8, 1:7, 1:6, 1:5, 1:4, 1:3.5, 1:3, 3:7, 1:2, 4:6, 5:5, 6:4, 7:3, 8:2, and 9:1. The pseudoternary diagrams were developed based on the aqueous titration method. Each oil and Smix underwent a slow titration with the aqueous phase while being moderately stirred. Water was added in varying amounts, ranging from 5 to 95% of the total volume. The formulation was visually observed after the addition of the water to the volume [19].

### 4.6. Preparation of the Nanoemulsion

Using Timur–rosemary oil as the oil phase and distilled water as the aqueous phase, the nanoemulsion was formulated. Under continuous stirring at high speed using a vortex mixer at room temperature, rosemary oil was dissolved into the oil phase at room temperature. After the drug had been fully dissolved in the oil phase, Smix (Tween 80 and Transcutol P) was introduced. Under continuous stirring, the water was added drop by drop into the oil phase. The mixture was then subjected to gentle agitation using the vortex for 3 min. The ideal formulation was selected based on the polydispersity index (PDI) and droplet size. Before evaluating each formulation’s droplet size, polydispersity index (PDI), and physical characteristics, each formulation was self-emulsified in distilled water with gentle agitation [55,56].

#### Physicochemical Characterization of Nanoemulsion

The developed formulations were characterized for their physicochemical parameters, droplet sizes, and PDIs. Equipment from Malvern Instruments was utilized to determine the particle size and PDI. The Timur–rosemary oil was prepared as a nanoemulsion using a titration process [57].

### 4.7. Fabrication of Timur Oil Nanoemulgel Formulation

The best formulation was chosen to be the nanoemulsion with the greatest concentration of Timur and rosemary oil, the smallest particle size, and the lowest PDI. The developed nanoemulsion formulation was selected for the fabrication in the nanoemulgel with different Carbopol Ultrez 21 and Carbopol 940 concentrations (viz., 0.5%, 1%, 1.5%, 1.8%, and 2% gels). First, Carbopol hydrogels were prepared using Carbopol 940 and Carbopol Ultrez 21 as thickening agents by dispersing Carbopol in purified water and left-over night for swelling, then the pH of the hydrogel was neutralized using 0.05% of triethanolamine (TEA), and DMDM was added as a preservative. Then, the hydrogel matrix was mixed with the developed nanoemulsion (5 mL) with 1% rosemary oil and 2% Timur oil at 100 rpm until the nanoemulgel was formed. In order to achieve uniformity, each formulation was thoroughly blended [58].

#### 4.7.1. Physical Characterization of Timur Oil Nanoemulgel Formulation

We visually assessed a wide range of physical characteristics, including consistency, spreadability, homogeneity, phase separation, and visual appearance, when developing the nanoemulgel. Using a pH meter, the pH values were determined (Mettler Toledo Inc., Columbus, OH, USA).

##### Zeta Potential

Equipment from Malvern Instruments was used to determine the zeta potential to predict the dispersion stability and surface charge of the particles. The zeta potentials were measured in triplicate [59,60,61,62,63].

##### Transmission Electron Microscopy (TEM)

Transmission electron microscopy (TEM) is a high-resolution technology that can be used to investigate specimens at the nanoscale. The developed formulation was studied for its surface morphology with the help of TEM, which was operated at 20–120 kV (Thermo Scientific Talos L120C G2 (S)TEM Microscope, Waltham, MA, USA) and carried out at Jamia Hamdard, New Delhi, India, enabling point-to-point resolution. A droplet of the sample was applied to a 300 mesh grid of copper-coated carbon that had been adequately saturated with water (1:1000), dyed with phosphotungstic acid (2% *w*/*v*), and allowed to air dry for about one minute before evaluation [54].

##### Mechanical Properties of NEG

To determine the gel’s mechanical attributes, including its hardness, cohesiveness, adhesiveness, gumminess, chewiness, and elasticity, a texture study was conducted. The texture analysis was performed using a texture analyzer (TA-XT Plus, Stable Microsystems, Godalming, UK). Using a probe that dips into the formulation at a certain speed and with a predetermined amount of force, data were collected and analyzed. A beaker free of air bubbles was filled with 100 g of the gel. A disc of Perspex (diameter 40 mm) was attached to the handle, and speeds of 1.0 mm/s before the test, 2.0 mm/s during the test, and 10.0 mm/s after the test were set. A 25 mm diameter cylinder probe was used for the penetration test, with a 5 mm penetration depth and a test speed of 2 mm/s in compression mode. Data computation was performed in Texture Exponent Software. All the tests were performed at room temperature using a triple (mean ± SD) set [62].

#### 4.7.2. In Vitro Release Study

The dialysis bag (Sigma, St. Louis, MO, USA) was kept in running water for 3–4 h for the removal of glycerine and then treated with a 0.3% *w*/*v* sodium sulfide solution in water at 80 °C for 1 min for the removal of sulfur compounds. It was then washed with hot water at 60 °C for 2 min. The procured dialysis bag was acidified with 0.2% *v*/*v* H_2_SO_4_ in distilled water. It was then rinsed with hot water to remove the acid and stored in the dissolution medium in the refrigerator to keep the pores open [63]. The essential oil release from antifungal nanoemulgel was determined in phosphate buffer at pH 5.5 using the dialysis bag method [54,64]. 

A freshly prepared formulation (0.5 gm of gel) was put in the dialysis bag (MWCO 12KD, Sigma), pre-soaked in double-distilled water for 12 h before use, and sealed. The dialysis bags were put in a beaker containing 100 mL of phosphate buffer at pH 5.5, which acted as a receptor compartment; it was maintained at 37 °C and stirred at a speed of 150 rpm. At an appropriate time interval (0.5, 1, 2, 4, 6, up to 24 h), a 3 mL aliquot of the dissolution medium was withdrawn, and the same volume of fresh dissolution medium was then added. A good sink condition was maintained throughout the test. The amount of the drug was evaluated using a UV spectrophotometer at 272 nm for the developed formulation [63].

%Cumulative drug release = concentration(µg/mL) × volume of dissolution media(mL) × Dilution Factor × 100/Initial amount of drug.

##### Release Kinetics

The data from the drug release studies were fitted with different kinetic models, such as the zero-order model, first-order model, Higuchi matrix model, and Korsmeyer–Peppas model. The correlation coefficient (R^2^) for each model was calculated using the following formula. The model resulting in the R^2^ closest to 1 was chosen as the best fit for drug release [65].

Zero order model Q = kt First order model log Q = kt/2.303 Higuchi model Q = k√t Korsmeyer–Peppas model = kt^n^

#### 4.7.3. Antifungal Activity of Formulations

The antifungal activity of the rosemary oil, Timur oil, lavender oil (standard), ketoconazole (standard), developed nanoemulsion, and developed nanoemulgel with regard to *C. albicans* was determined following the method demonstrated by Kadimi et al. [66]. A McFarland standard was used for preparing the suspension of the organism. The organism was first grown on sabouraud dextrose agar medium (pH 6.2). The medium was prepared and sterilized in an autoclave for 20 min at 121 °C. A sterile borer was used to create wells that were 6 mm in diameter. After 24 h of incubation, the inoculum was firmly swept over the agar plate using a sterile cotton swab to make uniform culture lawns. Quantities of 0.1 gm of different gels (0.5, 1%, 1.5%, 1.8%, and 2%) with different concentrations of Timur oil and rosemary oil were poured into wells with the help of a sterile spatula and incubated for 48 h, and these plates were assessed for clear zones around the wells. The zones of inhibition were assessed by measuring the diameters [67].

##### Calculation

Based on the ZOIs of the control and test samples, the % inhibition was calculated using the formula below:

% inhibition = AIC − AIT/AIC × 100

where AIC is the area of inhibition for the control and AIT is the area of inhibition for the extracts.

#### 4.7.4. Ex Vivo Permeation Study

Male Wistar rats (weighing between 150 and 200 g) were acquired from Institutional Animal Ethical Committee constituted by Jamia Hamdard, under protocol number 1575, which was utilized to prepare the skin for permeation testing. The rats were euthanized by CO_2_ inhalation. The skin was removed from the area of the dorsal. With the use of “veet cream”, the hairs were plucked. Using isopropyl alcohol and a cotton swab, the fat was eliminated [68]. The skin was then cleaned with distilled water and kept at −21 °C for scientific investigation. The skin penetration study was conducted on the dorsal skin of a rat used as the model skin for the Franz diffusion cell. The sample skin was positioned between the donor compartment and the receptor compartment, with the stratum corneum towards the donor compartment. The sample formulation served as the donor medium, while phosphate-buffered saline served as the receptor (pH 5.5). After 15 min, 30 min, 1 h, 2 h, 4 h, 6 h, and 24 h, the receptor medium (1 mL) was removed and replaced with a new medium. After the dilutions, a 0.45 µm membrane filter was used to filter the solutions, and a UV spectrophotometer was used to measure the quantity of drug in the receptor medium [69].

##### Data Analysis

After the study, the flux was plotted against time on a graph. The quantity of the drug that permeated was determined using the formula:

Amount of drug permeated (µg/cm^2^) = concentration (µg/mL) × volume of dissolution media/Area (cm^2^)

The volume of dissolution media was equal to 35 mL, and the area of the diffusion cell was equal to 1.966 cm^2^.

The flux was equal to the slope of the steady-state portion of the plot between the amount of drug permeated per cm^2^ vs. time in µg/cm^2^/h.

The permeability coefficient (Kp) was equal to flux/initial drug concentration (C_0_).

#### 4.7.5. Drug Deposition Study

The quantity of drug held in the skin sample used in permeation testing was used to measure the capacity of the globules to help keep the drug inside the skin layers. The skin placed on the diffusion cell was taken off once the permeation experiment was finished. In order to remove any adherent product, the skin was cleansed with cotton soaked in ordinary saline solution and wiped with tissue paper. In order to extract the medication, the skin sample was then homogenized with 10 mL of phosphate buffer at pH 5.5. The resulting homogenate solution was filtered through a membrane filter (0.45 µm), and its drug content was measured using a UV spectrophotometer at 272 nm. All the readings were collected three times for accuracy [70].

#### 4.7.6. Confocal Laser Scanning Microscopy (CLSM)

For confocal microscopy, gels were applied to the dorsal skin of the rats. Formulations containing the vesicles loaded with the fluorescent dye rhodamine were prepared by adding the dye (as a marker) to the nanoemulgel. Confocal pictures were acquired as XY-planes to show the fluorescence distribution (parallel to the plane of the skin surface). The brightest fluorescence with a morphology of stratum corneum surface resembles the skin surface in the imaging plane. The process of creating an XZ-section involves drawing a horizontal line across the area of interest in the Z = 0 µm XY-plane. This line is then “optically sliced” across the digital image data of the subsequent XY sections, yielding (XA-planar) optical cross-sections. All the photos were produced using an identical optical aperture, lens, and scan speed, and were averages of the triplet scans. The CLSM was used to examine the extent and mechanism of the rhodamine-loaded nanoemulgel’s skin permeability [71,72].

#### 4.7.7. Skin Irritation Studies

This study was carried out on healthy Wistar rats weighing 150–200 gm. By performing a Draize patch test, the irritability potential of the antifungal nanoemulgel was compared with that of the commercialized antifungal cream [73,74,75]. The protocol (1575) was approved by the Institutional Animal Ethical Committee constituted by Jamia Hamdard for such a purpose. Throughout the whole experimental process, animals were cared for and handled in accordance with CPCSEA criteria. Experimental Wistar rats were used to assess the irritability of the developed nanoemulgel (Tim–Ros–NEG) [76]. With care taken not to harm the skin’s top layer, the hair from the dorsal side of the acclimatized animals was removed 24 h before the experiment. The animals were subsequently split into five groups (*n* = 3), with the first group (Group I; the control group) receiving no treatment (no drug treatment) and Group II received 0.8% (*v*/*v*) aqueous formalin solution as a standard irritant. Group III, Group IV, and Group V received the developed nanoemulgel (1% rosemary oil + 2% Timur oil), developed nanoemulsion (1% rosemary oil + 2% Timur oil), and marketed antifungal preparation, respectively. In the drug-free treatment group, an equal amount of blank NEG was applied to the cleansed skin across a 1 cm^2^ area instead of the formulations. At 24, 48 and 72 h, the skin of the test animals was examined for any dermal responses, namely erythema or edema [73].

#### 4.7.8. Histopathology of Skin

Histopathology studies were performed for the evaluation of the rat skin for any damage resulting from the application of the various drugs on the skin surface. The animals were sacrificed after one week, and the skin was removed from the interscapular region using scissors and forceps. Samples were prepared and sectioned by utilizing a microtome. Furthermore, hematoxylin and eosin dye were used to stain the sectioned samples. The specimens were then examined using a high-power microscope to assess their integrity and perform stratum corneum and epidermal examinations. The results were compared with those of the rats in the control group [77].

#### 4.7.9. Statistical Assessment

The data were acquired in triplicate for each of the established experiments, and the values were presented as mean ± S.D. Statistical significance was considered when the *p*-value was less than 0.05.

## Figures and Tables

**Table 1:** Observed wavenumbers of different functional groups in the sample.

Sample No.	Functional Group	Wavenumber (cm^−1^)	Observed (cm^−1^)
1	OH stretching	3550–3200	3399.83
2	C-H Stretching	3000–2840	2923.29
3	C=C Stretching	1680–1630	1640.34
4	C-H bending	1600–1400	1451.66
5	C-O stretching	1124–1087	1090.89

**Table 2:** GC analysis of Timur oil.

Parameter	Linalool Content in Timur Oil (Nepali Variety)	Standard (Linalool)
Area	51,550,363	272,960,345
Retention time	15.531	15.583

**Table 3:** Different nanoemulgel formulations with different polymer and drug concentrations.

Formulation Code	Drug Concentration		Carbopol 940 (% *w*/*v*) Concentration	Particle Size (nm)	Resultant PDI	ZOI (mm)
	Timur Oil (%)	Rosemary Oil (%)				
FA	1	2	0.5	255	0.590	9
FB	0.5	0.5	1.0	195	0.521	10
FC	1.0	1.0	1.5	166	0.412	11
FD	2.0	1.0	1.8	139	0.309	15
FE	1.0	0.5	2.0	172	0.389	10

**Table 4:** Texture analysis of developed formulation.

Test ID	Batch		Firmness (g) Force 1	Consistency (g. s) Area F-T 1:2	Cohesiveness (g) Force 2	Index of Viscosity (g. s) Area F-T 2:3
Start Batch Force	Force					
Force 1	Force		783.01	898.34	−459.81	−688.84
End Batch Force	Force					
Average:	Force (F)	AVERAGE (“BATCH”)	783.01	898.34	−459.81	−688.84
S. D.	Force (F)	STDEVP (“BATCH”)	0.00	0.00	0.00	0.00
C. V.	Force (F)	STDEVP (“BATCH”)/AVERAGE(“BATCH”) ×100	0.00	0.00	0.00	0.00
End of Test Data						

**Table 5:** Drug retention of pure Timur oil and nanoemulgel on rat skin.

The Dose Applied to the Skin	Drug Deposited (µg)
1 g of nanoemulgel	56.45
1 mL of pure Timur oil	34.56

**Table 6:** Mean erythema scores for various formulations obtained at the end of 24, 48 and 72 h.

Formulations	Erythyma Score (Total)		
	24 h	48 h	72 h
Control (Group I)	0	0	0
Aqueous formalin solution (Group II)	3	4	4
Developed nanoemulgel (1% rosemary oil + 2% Timur oil) (Group III)	0	0	0
Developed nanoemulsion (1% rosemary oil + 2% Timur oil) (Group IV)	0	0	0
Marketed gel (Group V)	1	2	2

**Table 7:** Assessment of antifungal activity of samples against *C. Albicans*.

S. No.	Disk Code	Formulations/Samples	Concentration (µg/mL)	ZOI (mm)
1.	5 ^a^	Smix	10	00 ± 00
2.	6 ^a^	Timur oil	10	11 ± 0.7
3.	7 ^a^	Lavender oil	10	5 ± 0.9
4.	8 ^a^	Rosemary oil	10	9 ± 0.5
5.	5 ^b^	Placebo nanoemulgel	10	0 ± 0.0
6.	6 ^b^	Timur:rosemary (2:1)	10	10 ± 0.4
7.	7 ^b^	Timur oil	10	11 ± 0.7
8.	8 ^b^	Developed Nanoemulsion	10	16 ± 1.01 *
9.	5 ^c^	Placebo nanoemulsion	10	0 ± 0.0
10.	6 ^c^	Ketoconazole (standard)	10	13 ± 0.8
11.	7 ^c^	Timur:rosemary (2:1)	10	10 ± 0.4
12.	8 ^c^	1.8% nanoemulgel	10	15 ± 2.9

^a^ Figure 15A; ^b^ Figure 15B; ^c^ Figure 15C. An asterisk (*) indicates that the value is significantly different at *p* < 0.05 compared to the other formulations.

## Data Availability

The data presented in this study are available on request from the corresponding author.
